# Nano-selenium Supplementation Increases Selenoprotein (Sel) Gene Expression Profiles and Milk Selenium Concentration in Lactating Dairy Cows

**DOI:** 10.1007/s12011-020-02139-2

**Published:** 2020-04-23

**Authors:** Liqiang Han, Kun Pang, Tong Fu, Clive J. C. Phillips, Tengyun Gao

**Affiliations:** 1 College of Animal Science and Veterinary Medicine, Henan Agricultural University, Zhengzhou, 450002 China; 2 College of Animal Science and Veterinary Medicine, Xinyang Agriculture and Forestry University, Xinyang, 464000 China; 3 Centre for Animal Welfare and Ethics, School of Veterinary Science, The University of Queensland, Gatton Campus, Gatton, 4343 Australia

**Keywords:** Milk, Nano-Se, Glutathione peroxidase, Selenoprotein, Dairy cow

## Abstract

Supplementation with selenium is common for dairy cows, but the importance of selenium source is not clear. This study aimed to compare nano-selenium (Nano-Se) and sodium selenite supplements for dairy cows on lactation performance, milk Se levels and selenoprotein (Sel) gene expression. Twelve multiparous Holstein cows were randomly divided into two groups: a control group fed a basal diet plus 0.30 mg Se/kg of DM as sodium selenite or Nano-Se for 30 days. Dry matter intake, milk yield and composition were not affected by dietary Se source (*P >* 0.05); however, the milk total Se levels and milk glutathione peroxidase (GSH-Px) activities were higher with Nano-Se supplementation than sodium selenite (*P* < 0.05). At the end of the experiment, Nano-Se supplementation significantly increased plasma Se levels and GSH-Px activity, compared with the sodium selenite supplement. The mRNA expression levels of glutathione peroxidase 1, 2 and 4; thioredoxin reductase 2 and 3; and selenoproteins W, T, K and F were markedly upregulated (*P* < 0.05) in the mammary gland of the Nano-Se group. Thus, the source of selenium plays an important role in the antioxidant status and in particular the Sel gene expression in the mammary glands of dairy cows, both being stimulated by nano sources.

## Introduction

Selenium (Se) is a trace element nutrient that acts as a cofactor of antioxidant enzymes (such as glutathione peroxidases) and affects the antioxidant activities and immune functions of animals [1]. Se deficiency may cause immune system or nerve damage and several diseases in animals [2, 3]. Se supplementation has antioxidant effects, thereby increasing cellular defence against oxidative stress [4, 5]. Free radicals damage cells and limit the bactericidal effects of a neutrophil release. Therefore, Se is essential for the health of animals, including humans for whom an allowance of ≥ 55 μg/day decreases the risk of cancer [6].

Milk potentially makes an important contribution to a person’s daily intake of Se, as consumption of 100 g milk/day will provide at least 10% of the daily Se requirement for adults [7]. Therefore, supplements of dietary Se to increase milk Se content have been developed in cows [8]. Early studies found that after feeding inorganic forms of Se, such as sodium selenite, small amounts of Se were transferred to milk [9]. Dietary organic Se (Se yeast) have also resulted in increased Se concentrations in blood and milk, associated with a reduction in oxidative stress status in cows [10–13].

Nano-selenium (Nano-Se) is a new method of supplementing with Se that uses a protein as a dispersant and the red element Se as a membrane. Nano-Se contains more active centres than conventional Se products, with higher biological activity and lower toxicity, and is more effective at increasing selenoprotein (Sel) expression [14–16]. Elemental Se is biologically inert, but supplements with nanometre dimensions, typically 20–60 nm, may have improved absorption characteristics. For example, it has been reported that Nano-Se supplementation had higher Se deposition efficiency than sodium selenite in laying hens [15]. Information on the effects of Nano-Se supplementation on bovine lactation performance and milk Se is limited. Therefore, the aim of this work was to evaluate the effects of Nano-Se supplementation on lactation performance, milk Se concentration and mammary Sel expression in mid-lactation dairy cows in comparison with conventional sodium selenite.

## Materials and Methods

### Ethics Statement

All experimental procedures were approved by the Animal Care and Use Committee of Henan Agricultural University, which was performed according to the Guidelines for Experimental Animal of the Ministry of Science and Technology (2006, Beijing, China).

### Diets and Feeding

Twelve multiparous Holstein cows (2.5 ± 0.3 parity, 141 ± 27 days in milk [DIM], 583 ± 34.6 kg BW, 28.6 ± 2.4 kg milk/day) were randomly divided into two groups with six cows in each group. Treatment diets consisted of a basal diet plus 0.30 mg Se/kg DM from sodium selenite (analytical grade, 1% Se content, Xingjia Bio-Engineering Co., Ltd., Changsha, China) for the control group and a basal diet plus 0.30 mg Se/kg DM from Nano-Se (0.01% Se content, Bosar Biotechnology Research Co. Ltd., China) for the Nano-Se group. The experimental cows were fed a total mixed ration (TMR). The experimental period was 35 days, with 5 days of adaptation and 30 days of sampling. Cows were housed in a naturally ventilated barn, fed individually in their own troughs. They were milked three times daily at 6:00, 14:00 and 20:00 h and were fed ad libitum a total mixed ration (TMR) after each milking. The ingredients and chemical composition of the diets are reported in Table 1. Rations were formulated to meet the cow’s predicted requirements for minerals and vitamins according to the National Research Council (NRC, 2001).

**Table 1:** Ingredients and chemical composition of the basal diets of cows

Composition	Content
Ingredient, in g/kg DM	
Corn silage	420
Ground corn	38
Steam-flaked corn	155
Soybean meal	85
Cottonseed	40
Oat hay	43
Alfalfa hay	132
Soybean hull	54
Yeast XP^1^	4.2
NaCl	3.8
Limestone	5.8
NaHCO_3_	6.2
KHCO_3_	5.4
MgO	2.6
Premix^2^	5
Chemical composition, in g/kg DM	
Crude protein	167
Neutral detergent fibre	315
Acid detergent fibre	208
Ether extract	56
Net energy of diet, Mcal/kg^3^	1.81
Ca	8.1
P	4.5
Se (mg/kg DM)	0.05

^1^Purchased from Diamond V Co., USA

^2^Composition in kg^−1^ DM: 670,000 IU of vitamin A; 92,000 IU of vitamin D; 3750 IU of vitamin E; 700 mg of niacin; 2000 mg of Cu; 4000 mg of Zn; 330 mg of Mn; 65 mg of I; 37 mg of Co.

^3^Calculated using NRC

### Milk Sampling and Feed Dry Matter Intake Recording

Feed offered and refused were measured daily for each cow and recorded throughout the experimental period to calculate DM intake. Milk production was recorded daily, and consecutive morning, midday and evening samples were collected every 7 days (day 1, day 7, day 14, day 21, day 28). Milk samples were divided into two, one stored with preservative at 4 °C for the analysis of fat, protein and lactose by infrared spectrophotometry (Foss 120 Milko-Scan, Foss Electric, Denmark), and the second stored at − 80 °C for later chemical analysis. Samples of TMR were collected once per week and stored at − 20 °C for later chemical analysis.

### Blood Samples

Blood samples were collected from the coccygeal vein before feeding using evacuated tubes containing heparin on the 30th day of the experiment. After blood collection, plasma was obtained by centrifugation at 2000×*g* for 30 min at 4 °C. Aliquots of plasma were frozen (− 80 °C) until further analysis.

### Chemical Analyses

The concentrations of Se in feed sample, milk and plasma samples were analysed by the fluorometric method [11]. Briefly, the samples were digested with perchloric acid and nitric acid. After adding hydroxylamine and EDTA solutions to the digestive solution, the pH value of the mixed solution was adjusted to 1.5. Then, a 2,3-diaminonaphtalene solution was added. The resulting solution was incubated in a boiling water bath for 5 min in darkness and extracted with cyclohexane after cooling. The fluorescence intensity of the organic phase was determined by a fluorescence spectrophotometer (Hitachi F-7000, Japan), and the Se concentrations of the samples were quantified with external calibration using a regression equation.

Milk samples were centrifuged at 10,000×*g* for 30 min at 4 °C, after which the upper layer of milk fat was removed and the resultant milk whey was diluted 1:10 with PBS to detect the GSH-Px enzyme activity. The GSH-Px activity of both the milk whey and plasma was measured using a glutathione peroxidase (GSH-Px) assay kit (A005, colorimetric method, from Nanjing Jiancheng Bioengineering Institute) according to the manufacturer’s instructions.

### Sel Gene Expression in the Mammary Gland

A biopsy sample of mammary gland tissue (*n* = 5) was obtained percutaneously on day 30, as described previously [17]. Total RNA was isolated from 50 to 60 mg of mammary gland tissue using an miRNeasy kit (Qiagen) following the manufacturer’s protocols. Samples were treated with DNaseI (Qiagen) and quantified using a NanoDrop ND-1000 (NanoDrop Technologies). First-strand cDNA was synthesized using Oligo(dT)20 and Superscript II reverse transcriptase. A comprehensive literature search [18–22] was conducted to select the Sel genes to be evaluated, including glutathione peroxidase1~4 (GPX1~4), thioredoxin enzyme1~3 (TXNRD1~3) and Selenoprotein gene (M, W, F, P, etc.). Primers were designed according to the NCBI database using the *Bos taurus* sequence (https://www.ncbi.nlm.nih.gov/) to produce PCR products. Gene symbols and primer sequences are presented in Table 2. Real-time PCR was then performed as previously described [17]. The relative mRNA expression of the target gene was normalized with the geometric mean of UXT, RPS9 and RPS15 [23].

**Table 2:** Primer sequences of Sel genes for real-time PCR

Gene symbol (GenBank number)	Primer sequence (5′-3′)	Length of products	Gene name
GPX1 (NM_174076)	F: AACGTAGCATCGCTCTGAGG R: GATGCCCAAACTGGTTGCAG	121	Glutathione peroxidase 1
GPX2 (NM_001163139)	F: TGAGCATTCACTGTGCCCTC R: GGAAGGAAACAGGCAGACCA	104	Glutathione peroxidase 2
GPX3 (NM_174077)	F: TTGGTCTGGTCATTCTGGGC R: CCCCACCTGGTCGAACATAC	105	Glutathione peroxidase 3
GPX4 (NM_174770)	F: CCGAGATGAGCTTTAGCCGT R: TGGCTGAAAATTCGTGCATGG	144	Glutathione peroxidase 4
TXNRD1 (NM_174625)	F: CATTGCCACTGGTGAAAGGC R: CCAACCACCAGGGTCTTACC	117	Thioredoxin reductase 1
TXNRD2 (NM_174626)	F: ACCACGTGAAGTCCCTGAAC R: CCTTTGGAGACACCGCAAAC	118	Thioredoxin reductase 2
TXNRD3 (NM_001192109)	F: CAGCACGCGGGTTAAAGAAC R: CTGGTGATCTCCGACAGAGC	114	Thioredoxin reductase 3
SelM (NM_001163171)	F:ACTGGAACCGTCTACAAGGC R:GGATGTCCTGGGTGACGAAG	105	Selenoprotein M
SelT (NM_001103103)	F: TTTTGCAAGAGCAGCGTGAC R: AAGAGGTACAACGAGCCTGC	102	Selenoprotein T
SelW (NM_001163225)	F: CTAGCCGTCTGGACATCTGC R: TGGAGTGAACCAGCTTTCCC	87	Selenoprotein W
SelH (NM_001164092)	F: GCTTCGAGGTGACGTTGCTG R:CTGCCCCACCTCCCTACGA	150	Selenoprotein H
SelK (NM_001037489)	F:AGGCTACGGAAGCTCATCTG R: CGGCCATTGGAGGAGGATTA	122	Selenoprotein K
SelI (NM_001075257)	F: ACAAGCATGTACCCGACTGG R: GAATTGGTTCTGCGGGCTTG	103	Selenoprotein I
SelO (NM_001163193)	F: CCACGTTCCTCAGGTTTGGA R: ATCTGCAGTCGGATGTCGTC	103	Selenoprotein O
SelS (NM_001046114)	F: CCCACCCTCGAGACCGA R: ATGTACCAGCCGTAAGTGGC	77	Selenoprotein S
SelF (NM_001034759)	F: TTGGGGAGGTTCCCTCAAGT R: AGCAATGTTCCCACTGTCGT	132	Selenoprotein F
SelV (NM_001163244)	F: CCATCCAGGCCATCTTACCG R: AGGCCACAGTAAACCACTCG	103	Selenoprotein V

### Statistical Analysis

Data statistics and differences among groups were analysed by SPSS 25.0 (SPSS/IBM Corp., Chicago, IL). The data describing DM intake, milk yield, milk composition and milk Se were analysed for significant differences using a repeated measures general linear model. The model included the factors of diet (*D*), time (*T*) and their interactions (D × T). Differences between means were tested using the LSD test. GPX-PX activity, blood parameters and gene expression were analysed using Student’s *t* tests. A *P* value < 0.05 was considered statistically significant.

## Results

### Lactation Performance

There were no differences between treatments, dates or interactions between the two (*P* > 0.05) for cow DMI, milk yield, milk protein, fat or lactose concentration (Table 3). However, Nano-Se supplementation led to greater milk Se than the control treatment (+ 7.38 μg/L kg/d, *P* < 0.01; Table 3).

**Table 3:** Effects of Nano-Se supplementation on lactation performance and milk composition of dairy cows (*n* = 6)

	Control	Nano-Se		*P* value		
Item			SEM	Treatment	Time	Treat × time
DMI (kg/day)	13.45	13.28	0.35	0.74	0.17	0.79
Milk yield (kg/day)	26.74	27.44	0.64	0.36	0.11	0.19
Milk protein (%)	3.34	3.50	0.10	0.31	0.63	0.32
Milk fat (%)	3.37	3.33	0.22	0.87	0.79	0.80
Milk lactose (%)	4.87	4.86	0.05	0.94	0.66	0.67
Milk Se (μg/L)	23.19	30.60	1.55	< 0.01	< 0.01	< 0.01

### GSH-Px Activity in Milk

**Fig. 1:**
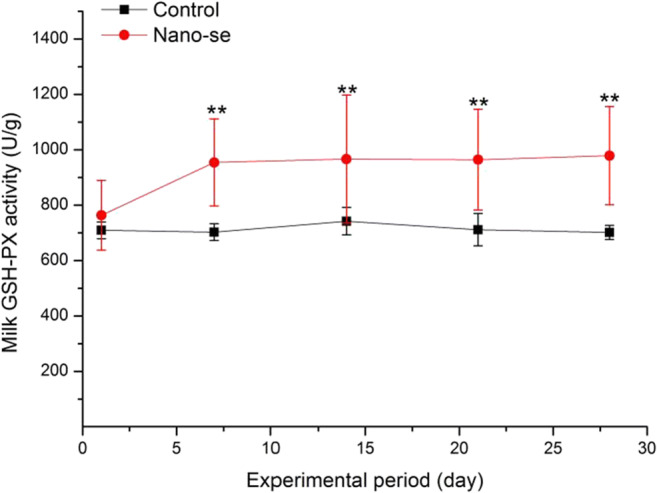
Effect of Nano-Se supplementation on the milk GSH-Px activity. Data are presented as the mean ± SEM. The milk samples were collected on day 1, 7, 14, 21 and 28. One unit of GSH-Px is defined as the amount of enzyme depleting 1 μmol of GSH per gram of milk protein /5 min at 37 °C. *P < 0.05 or **P < 0.01

The milk GSH-Px activity is presented in Fig. 1. Milk in the Nano-Se group had higher GSH-Px activity than the control treatment at days 7, 14, 21 and 28 (*P* < 0.05 or *P* < 0.01).

### Antioxidant Capacity in Blood

Compared with controls, cows in the Nano-Se treatment had increased plasma Se and plasma activity of GSH-Px (*P* < 0.01, Table 4).

**Table 4:** Effects of Nano-Se supplementation on the antioxidant activity of plasma (*n* = 6)

Item	Control	Nano-Se	SEM	*P* value
Total Se (μg/L)	110.13	137.49	5.89	< 0.01
GSH-Px activities (U/mL)^1^	131.20	161.53	6.49	< 0.01

^1^One unit of GSH-Px is defined as the amount of enzyme depleting 1 μmol of GSH /5 min at 37 °C in 0.1 mL of serum

### Sel Gene Expression in the Mammary Gland

Compared with controls, Nano-Se supplementation significantly increased mRNA expression of GPX1, GPX2, GPX4, TXNRD2, TXNRD3, SelW, SelT, SelK and SelF in the mammary gland. Expression of mRNA of other Sel genes (GPX3, TXNRD 1, SelM, SelH, SelI, SelO, SelS and SelV) was not affected by the Nano-Se treatment (*P* > 0.05, Table 5).

**Table 5:** Effects of Nano-Se supplementation on the mRNA expression of Sel genes in the mammary gland of dairy cows (*n* = 5)

Gene	Control	Nano-Se	SEM	*P* value
GPX1	0.64	1.54	0.12	< 0.01
GPX2	0.82	1.58	0.07	< 0.01
GPX3	1.18	1.39	0.16	0.33
GPX4	0.49	1.52	0.08	< 0.01
TXNRD1	0.91	1.32	0.25	0.21
TXNRD2	0.63	1.64	0.24	0.02
TXNRD3	0.90	1.25	0.06	< 0.01
SelM	1.27	1.23	0.25	0.91
SelW	0.90	1.20	0.54	0.02
SelT	0.90	1.58	0.12	< 0.01
SelH	0.88	1.29	0.09	0.19
SelK	1.01	1.52	0.12	0.01
SelI	0.97	1.11	0.05	0.06
SelO	0.84	1.00	0.08	0.16
SelS	1.05	1.32	0.11	0.06
SelF	0.84	1.31	0.18	0.04
SelV	1.01	1.05	0.04	0.51

## Discussion

In the present research, the Se concentration in the basal diet was measured as 0.05 mg/kg DM, which is the critical level for Se deficiency in cattle. A meta-analysis has indicated that cows supplemented with Se yeast (6 mg/head) each day had increased milk Se levels [9]. As an indicator of Se status in cows, concentrations of serum Se between 80 and 160 μg/L have been reported as adequate [24]. In the present study, we used a dose of 0.30 mg Se/kg DM, which meant that cows fed the sodium selenite and Nano-Se received 6.4 mg and 6.2 mg of Se/head, respectively. The plasma Se of control and Nano-Se groups (110.13 μg/L vs 137.49 μg/L) was within normal limits.

The scientific literature on the effects of Se on lactation performance is inconclusive. Several studies found that dietary Se has no significant effect on milk yields and components [25, 26]. On the contrary, other studies found that Se may result in lower milk protein [27], decreased milk fat [13] and higher milk yields [28]. These results could be due to differences in Se forms and doses/or the composition of the diet. The present research supports a general view that dietary Se sources are unlikely to significantly affect milk yield or composition [11].

Increased serum GSH-Px activity with Nano-Se supplementation has been reported in laying hens [15]. Similarly, in the current experiment, Nano-Se increased both Se levels and GSH-Px activity in blood compared with sodium selenite supplementation. As an essential component of the antioxidant system, it seems likely that plasma Se enhanced the antioxidant capacity of animals. GSH-Px is important for Se-containing enzymes in the blood of animals, which function as an antioxidant by reducing hydrogen peroxide to water and catalysing the reduction of thioredoxin. GSH-Px activity has been widely adopted as an antioxidant biomarker for oxidative stress in dairy cows [11]. It is clear that cows supplemented with Nano-Se had signs of increased antioxidant status of dairy cows.

Our results showed that Nano-Se was more effective than inorganic Se at increasing the milk Se concentration. A research with broiler chickens has also demonstrated that nanoparticles are more effectively absorbed in comparison with the usual dietary additive, sodium selenite [29]. The difference between Nano-Se and sodium selenite was probably related to the different absorption processes. Interestingly, we also observed that Nano-Se supplementation increased the GSH-Px activity of milk whey. GSH-Px is the only enzyme containing Se in milk and its activity is mainly dependent upon the concentration of Se [30]. Therefore, it is not surprising that Nano-Se supplementation increased milk Se, accompanied by increased GSH-Px activity in milk. The antioxidant capacity of milk and dairy products is mainly due to Se and Se-containing enzyme [31]. The increase in milk Se content and GSH-PX activity that we detected suggests that antioxidant activity of milk could be enhanced by Nano-Se supplementation.

Se exists in the form of selenoproteins (Sels) that play important biological function in dairy cows [13]. The upregulated GPX mRNA expression and GSH-PX activity that we detected indicated that Sel expression of mammary regulated the activities of milk Se-containing enzyme. It has been reported that the Se levels play a regulatory role for Sels, and Sel expression in animal tissues and cells were induced by Selenium supplement [32–34]. Since in the present study, nano-Se supplement resulted in significantly upregulated Sel mRNA expression (GPX, TXNRD, SelW, T, K and F), it is suggested that Se status is an important regulator of selenoprotein activity and expression in the mammary gland of dairy cows.

## Conclusion

Nano-Se was more effective than sodium selenite at improving antioxidant status and increasing milk Se levels of dairy cows. The Sel expression was increased in the mammary gland tissue of dairy cows with Nano-Se supplement when compared with sodium selenite supplementation. The present findings provide initial evidence of benefits of Nano-Se supplementation in dairy cows.
